# Novel triphenylphosphonium-hydrazone salts: integrated experimental and computational insights into AChE inhibition and resistance-overcoming antimicrobial and antibiofilm potential

**DOI:** 10.1007/s00210-026-05002-8

**Published:** 2026-01-20

**Authors:** Metin Yıldırım, Hakan Ünver, Adem Necip, Büsra Hord, Mehmet Ersatir

**Affiliations:** 1 Department of Biochemistry, Faculty of Pharmacy, Harran University, Sanliurfa, Türkiye; 2 Department of Chemistry, Faculty of Science, Eskisehir Technical University, Eskisehir, Türkiye; 3 Department of Pharmacy Services, Vocational School of Health Services, Harran University, Sanliurfa, Türkiye; 4 Faculty of Pharmacy, Harran University, Sanliurfa, Türkiye; 5 Department of Chemistry, Faculty of Art and Science, Cukurova University, Adana, 01330 Türkiye

**Keywords:** Triphenylphosphonium derivatives, Hydrazone-based compounds, Acetylcholinesterase inhibition, Carbapenem-resistant bacteria, Antibiofilm activity, Molecular docking

## Abstract

Neurodegenerative disorders and multidrug-resistant (MDR) bacterial infections represent two major and interconnected global health challenges. However, current therapeutic strategies are largely limited by single-target approaches, insufficient efficacy against biofilm-forming MDR pathogens, and the lack of multifunctional small molecules capable of addressing both neurodegeneration and bacterial resistance simultaneously. These limitations constitute a critical technical bottleneck in contemporary drug discovery and underscore the urgent need for innovative, dual-action therapeutic scaffolds. In this study, five novel triphenylphosphonium–hydrazone derivatives (1a–1e) were rationally designed, synthesized, and fully characterized by FT-IR, 1H/13C NMR, and HR-MS/MS analyses to overcome these challenges by integrating neuroprotective and antimicrobial functionalities within a single molecular framework. All synthesized compounds exhibited potent acetylcholinesterase (AChE) inhibitory activity, with IC₅₀ values ranging from 8.66 to 13.9 µM, highlighting their strong neuroactive profiles. Notably, compound 1b emerged as the most effective AChE inhibitor (IC₅₀ = 8.66 µM), underscoring its promise as a lead scaffold for the development of next-generation anti-Alzheimer agents. Beyond enzyme inhibition, the compounds demonstrated significant antibacterial efficacy against clinically relevant carbapenem-resistant pathogens. In particular, compound 1d showed the strongest activity against *Acinetobacter baumannii* and *Klebsiella pneumoniae*, with MIC values of 32 µg/mL and 64 µg/mL, respectively. Importantly, all derivatives (1a–1e) exhibited dose-dependent antibiofilm activity, achieving up to 83.4% biofilm disruption in *Acinetobacter baumannii* and 72.8% in *Escherichia coli* at 1024 µg/mL, directly addressing a major resistance-associated bottleneck in antibacterial therapy. Molecular docking studies provided mechanistic validation of this multifunctional design, revealing a strong binding affinity of compound 1d toward PBP1A (PDB: 6OWS) and the AcrB efflux pump protein (PDB: 6PT1), suggesting a previously unexplored dual antibacterial mechanism involving simultaneous inhibition of cell-wall biosynthesis and efflux-mediated drug resistance. Overall, this study introduces a novel triphenylphosphonium–hydrazone platform, establishes a clear structure–activity relationship, and highlights its potential as a multifunctional therapeutic strategy against both neurodegenerative disorders and MDR bacterial infections.

**Supplementary Information:**

The online version contains supplementary material available at 10.1007/s00210-026-05002-8.

## Introduction

Phosphine-derived ligands have emerged as a focal point in biomedical research due to their extensive range of biological activities, including antiviral, antioxidant, antifungal, antibacterial, anticarcinogenic, and antitumor effects (Meijboom et al. 2025; Wojtala et al. 2023; Ochiai et al. 2025). Among these, triphenylphosphine (TPP) has gained particular prominence as a mitochondria-targeting agent. This is primarily due to its strong lipophilic nature and its ability to carry a stable, delocalized positive charge once oxidized. These properties enable TPP to cross phospholipid membranes efficiently and accumulate selectively within mitochondria, attracted by the organelle’s inherently negative membrane potential. A common approach to harness this targeting ability involves the covalent linkage of TPP to pharmacologically active compounds through ester or amide bonds, facilitating mitochondrial localization and improving therapeutic precision. Such TPP-based delivery systems have proven highly effective in the development of targeted treatments and diagnostic platforms for a variety of diseases (Shakaroun 2021; Yousif et al. 2009; Aktaş et al. 2022). It has been established through previous studies that fluorine-containing alkyl phosphonates exhibit enhanced electrophilic activation due to the electron-withdrawing nature of fluorine. This enhancement leads to improved biological activity of the compounds (Zhang et al. 2024).

Hydrazones, a well-recognized subclass of Schiff bases, are characterized by the presence of a distinctive azomethine (–CH = N–) functional group, which plays a crucial role in mediating their chemical reactivity and biological interactions. Over the past decades, hydrazones have garnered significant attention in medicinal and pharmaceutical chemistry due to their broad spectrum of pharmacological activities and favorable drug-like properties. One of the key features contributing to the enhanced bioactivity of hydrazones is their ability to engage in hydrogen bonding and other non-covalent interactions with biological targets. Previous studies have demonstrated that hydrazone-like compounds exhibit high reactivity (Dou et al. 2024). A growing body of experimental and computational studies has highlighted the diverse biological profiles exhibited by hydrazone derivatives (Yıldırım et al. 2025a; Kadyan et al. 2025; Boora et al. 2025). These include, but are not limited to, analgesic, anti-inflammatory, antidepressant, antimicrobial, anticancer, and anticholinesterase activities. Notably, several hydrazone-based compounds have demonstrated potent enzyme inhibition, particularly against α-glucosidase, selective lysine acetyltransferase p300 inhibitors for cancer therapy, Lck/Src/KDR inhibitors, succinate dehydrogenase inhibitors, carbonic anhydrase, acetylcholinesterase (AChE), and butyrylcholinesterase (BChE)-enzymes that are critically involved in the pathophysiology of metabolic disorders, neurodegenerative diseases such as Alzheimer’s, and cancer. In addition to their enzyme-inhibitory properties, many hydrazone derivatives exhibit significant antioxidant and antibacterial effects, further underscoring their potential as multifunctional therapeutic agents. Taken together, the structural diversity, synthetic accessibility, and multifaceted bioactivity of hydrazones make them highly attractive candidates for drug discovery and development (Omidi and Kakanejadifard 2020; Verma et al. 2014; Tafere et al. 2025; Dai et al. 2024; Hui et al. 2024; Wu et al. 2024; Xie et al. 2019; Yan et al. 2024; Kumari and Kumar 2025a).

Carbapenems represent one of the most important classes of antibacterial therapeutic agents, exhibiting an extremely broad spectrum of activity against both aerobic and anaerobic bacteria (Zhuorong and Along 2025; Karampatakis et al. 2025). They are widely regarded as the first-line treatment for severe infections caused by pathogenic strains such as *Acinetobacter baumannii* (*A. baumannii*), *Klebsiella pneumonia* (*K. pneumonia*), and *Escherichia coli* (*E. coli*) (Karampatakis et al. 2025; Dubey et al. 2025; Nourbakhsh et al. 2025). However, in recent years, the inappropriate and excessive use of antibiotics has led to the emergence and rapid spread of carbapenem resistance, which poses a serious global public health threat. Due to the limited efficacy of existing antibiotics and the increasing prevalence of multidrug-resistant bacteria, which pose a significant threat to human health**,** there is an urgent need for the development and synthesis of novel compounds with potential antibacterial activity (Iovleva et al. 2025; Oladipo et al. 2025**).**

In this study, a series of novel triphenylphosphonium hydrazone salt derivatives were synthesized and thoroughly characterized. In addition to their AChE inhibitory properties**,** their antibacterial activities were evaluated against carbapenem-resistant strains of *A. baumannii***,** *K. pneumoniae*, and *E. coli*. Furthermore, molecular docking studies were performed to elucidate the interactions between the most active compounds and key bacterial target proteins involved in vital cellular processes.

## Materials and methods

### Materials and equipment

All reagents and solvents were of analytical grade and used without further purification. Tri-*p*-tolylphosphine, 4-(chloromethyl)benzaldehyde, 3-methoxybenzohydrazide, 3-hydroxybenzohydrazide, 3-bromobenzohydrazide, 3-chlorobenzohydrazide, and benzohydrazide were obtained from commercial suppliers and employed as received. The ^1^H, ^13^C, and ^31^P nuclear magnetic resonance (NMR) spectra of the synthesized compounds were recorded on an Agilent 400 MHz spectrometer using DMSO-d₆. solvent. High-resolution mass spectrometry (HR-MS) measurements were conducted on an Agilent 6545 Accurate-Mass QTOF-MS instrument operated in positive electrospray ionization (ESI⁺) mode, with DMSO as the solvent. AChE (EC 3.1.1.7, from Electrophorus electricus), acetylthiocholine iodide (ATChI), and 5,5′-dithiobis-(2-nitrobenzoic acid) (DTNB, Ellman’s reagent) were purchased from Sigma-Aldrich (St. Louis, MO, USA). Tacrine hydrochloride, used as a reference inhibitor, and all other chemicals were of analytical grade and obtained from Merck (Darmstadt, Germany). Deionized water was employed in the preparation of all aqueous solutions.

### Methods

#### Synthesis of (4-formylbenzyl)tri-p-tolylphosphonium chloride (Compound 1)

**Fig. 1:**
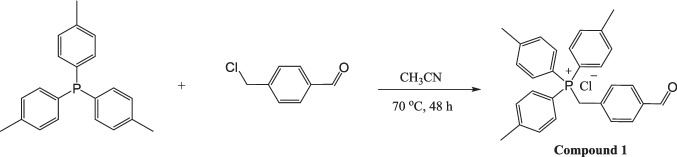
Synthesis procedure of (4-formylbenzyl)tri-p-tolylphosphonium chloride

Compound 1 was synthesized by reacting equimolar amounts of 4-(chloromethyl)benzaldehyde (154 mg, 1.0 mmol, 1.0 equiv) and tri-*p*-tolylphosphine (304 mg, 1.0 mmol, 1.0 equiv) in 20 mL of acetonitrile (CH₃CN). The reaction mixture was stirred at 70 °C until completion. Upon completion, the resulting precipitate was collected by filtration, washed with cold acetonitrile if necessary, and dried under vacuum. The crude product was used in the next step without further purification (see Fig. 1).

**Compound 1:** (305 mg, 67%).

^**1**^**H-NMR (400 MHz, DMSO-*d***_***6***_**):** *δ* (ppm) 9.93 (s, 1H), 7.79–7.72 (m, 2H), 7.59–7.44 (m, 12H), 7.19 (dd, J = 8.3, 2.5 Hz, 2H), 5.22 (d, J = 16.4 Hz, 2H), 2.43 (s, 9H). ^**13**^**C-NMR (100 MHz, DMSO-*d***_***6***_**):** *δ* (ppm) 193.11, 146.46, 146.43, 136.13, 136.10, 135.78, 135.70, 134.40, 134.30, 132.09, 132.03, 131.25, 131.12, 130.14, 130.11, 115.29, 114.41, 40.40, 29.36, 21.73, 1.61.

The syntheses scheme of (4-formylbenzyl)tri-p-tolylphosphonium chloride is given in Fig. 1.

#### Synthesis procedure of triphenylphosphonium-hydrazone derivatives

**Fig. 2:**
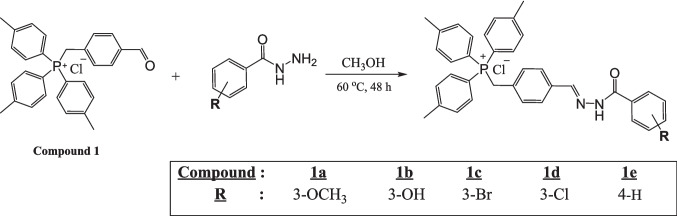
Synthesis procedure of triphenylphosphonium-hydrazone derivatives

A solution of Compound 1 (458 mg, 1.0 mmol, 1 equivalent) in 20 mL of methanol (CH₃OH) was prepared, and the appropriate *para*-substituted benzohydrazide (1.0 mmol, 1 equivalent) was added in portions. The reaction mixture was then stirred at 60 °C for 48 h (Fig. 2). After completion of the reaction, the mixture was allowed to cool to room temperature. The solid that formed was isolated by filtration and dried under vacuum.

**Compound 1a:** (81%).

^**1**^**H-NMR (400 MHz, DMSO-*d***_***6***_**):** *δ* (ppm) 12.01 (s, 1H), 8.47 (s, 1H), 7.51 (qd, *J* = 21.4, 18.7, 8.1 Hz, 16H), 7.10 (dd, *J* = 43.0, 7.5 Hz, 4H), 5.10 (d, *J* = 15.6 Hz, 2H), 3.82 (s, 3H), 2.45 (s, 9H). ^**13**^**C-NMR (100 MHz, DMSO-*d***_***6***_**):** *δ* (ppm) 163.31, 159.68, 147.40, 146.36, 134.41, 134.30, 131.79, 131.74, 131.22, 131.09, 130.12, 127.71, 120.37, 118.05, 115.47, 114.59, 113.38, 55.86, 39.79, 21.72. ^**31**^**P-NMR (162 MHz, DMSO-*d***_***6***_**):** δ (ppm) 22.23. **HR-ESIMS** calcd *m/z* for [M-Cl]^+^ C_37_H_36_N_2_O_2_P^+^ 571.6802, found 571.4141.

**Compound 1b:** (75%).

^**1**^**H-NMR (400 MHz, DMSO-*d***_***6***_**):** δ 11.83 (s, 1H), 9.78 (s, 1H), 8.39 (s, 1H), 7.64–7.42 (m, 14H), 7.29 (d, *J* = 16.8 Hz, 3H), 7.07–6.95 (m, 3H), 5.08 (d, *J* = 16.4 Hz, 2H), 2.45 (s, 9H). ^**13**^**C-NMR (100 MHz, DMSO-*d***_***6***_**):** *δ* (ppm) 146.40, 134.40, 134.30, 131.77, 131.71, 131.22, 131.09, 129.98, 127.73, 115.46, 114.97, 114.58, 21.72. ^**31**^**P-NMR (162 MHz, DMSO-*d***_***6***_**):** δ (ppm) 22.21 **HR-ESIMS** calcd *m/z* for [M-Cl]^+^ C_36_H_34_N_2_O_2_P^+^ 557.6532, found 557.3895.

**Compound 1c:** (69%).

^**1**^**H-NMR (400 MHz, DMSO-*d***_***6***_**):** 12.14 (s, 1H), 8.46 (s, 1H), 8.11 (s, 1H), 7.94 (d, J = 7.8 Hz, 1H), 7.80 (d, J = 8.1 Hz, 1H), 7.54 (td, J = 20.1, 8.4 Hz, 15H), 7.05 (d, J = 7.8 Hz, 2H), 5.11 (d, J = 15.8 Hz, 2H), 2.45 (s, 9H). ^**13**^**C-NMR (100 MHz, DMSO-*d***_***6***_**):** 162.06, 147.86, 146.39, 146.36, 135.85, 135.04, 134.57, 134.40, 134.30, 131.81, 131.75, 131.22, 131.09, 130.68, 127.37, 122.21, 115.46, 114.58, 21.73. ^**31**^**P-NMR (162 MHz, DMSO-*d***_***6***_**):** δ (ppm) 22.24 **HR-ESIMS** calcd *m/z* for [M-Cl]^+^ C_36_H_33_BrN_2_OP^+^ 620.5502, found 621.3119.

**Compound 1d:** (78%).

^**1**^**H-NMR (400 MHz, DMSO-*d***_***6***_**):** 12.10 (s, 1H), 8.44 (s, 1H), 7.97 (s, 1H), 7.89 (d, J = 7.8 Hz, 1H), 7.67 (d, J = 8.1 Hz, 1H), 7.53 (dq, J = 20.7, 9.3, 8.6 Hz, 15H), 7.05 (d, J = 7.8 Hz, 2H), 5.10 (d, J = 15.8 Hz, 2H), 2.45 (s, 9H). ^**13**^**C-NMR (100 MHz, DMSO-*d***_***6***_**):** 162.16, 147.84, 146.40, 134.40, 134.30, 133.75, 131.76, 131.22, 131.09, 131.00, 131.00, 127.84, 126.98, 115.45, 114.58, 40.00, 21.72. ^**31**^**P-NMR (162 MHz, DMSO-*d***_***6***_**):** δ (ppm) **HR-ESIMS** calcd *m/z* for [M-Cl]^+^ C_36_H_33_ClN_2_OP^+^ 576.0962, found 575.3586.

**Compound 1e:** (79%).

^**1**^**H-NMR (400 MHz, DMSO-*d***_***6***_**):** 12.07 (s, 1H), 8.48 (s, 1H), 7.94 (d, J = 7.6 Hz, 2H), 7.64–7.43 (m, 17H), 7.05 (d, J = 7.8 Hz, 2H), 5.11 (d, J = 15.9 Hz, 2H), 2.45 (s, 9H). ^**13**^**C-NMR (100 MHz, DMSO-*d***_***6***_**):** 163.58, 147.30, 146.38, 146.35, 134.74, 134.41, 134.31, 133.68, 132.30, 131.80, 131.74, 131.21, 131.09, 130.43, 128.92, 128.16, 127.71, 115.47, 114.60, 21.73, 21.72. ^**31**^**P-NMR (162 MHz, DMSO-*d***_***6***_**):** δ (ppm) 22.24 **HR-ESIMS** calcd *m/z* for [M-Cl]^+^ C_36_H_34_N_2_OP^+^ 541.6542, found 541.3978.

The syntheses scheme of triphenylphosphonium-hydrazone compounds is given in Fig. 2.

### AChE inhibition

Each compounds was dissolved in an appropriate solvent such as methanol or 0.1 M phosphate buffer (pH 7.4) to obtain a 1 mg/mL stock solution, and serial dilutions were prepared to achieve various working concentrations (1–8 µg/mL) for the enzyme inhibition assays. The inhibitory potential of the samples against AChE was determined spectrophotometrically according to the Ellman method (Ellman et al., 1961), with minor modifications to enhance sensitivity and reproducibility (Çimentepe et al. 2026). In a 96-well microplate, the reaction mixture contained 140 µL of 0.1 M phosphate buffer (pH 8.0), 20 µL of the test sample at the desired concentration, 20 µL of AChE enzyme solution (0.22 U/mL), and 10 µL of DTNB solution (0.5 mM). After preincubation for 15 min at 25 °C, the reaction was initiated by adding 10 µL of ATChI (0.71 mM). The increase in absorbance, resulting from the formation of the 5-thio-2-nitrobenzoate anion, was monitored at 412 nm for 10 min using a microplate reader (BioTek Synergy HT, USA). Controls (without inhibitor) and blanks (without enzyme) were included in each assay, and tacrine served as the positive control. All experiments were performed in triplicate, and results were expressed as mean ± standard deviation (SD).

The percentage inhibition of AChE activity was calculated using the equation: Inhibition (%) = [1—(As—Asb)/(Ac—Acb)] × 100, where *As* is the absorbance of the sample, *Asb* is the absorbance of the sample blank, *Ac* is the absorbance of the control, and *Acb* is the absorbance of the control blank.

The half-maximal inhibitory concentration (IC₅₀) values were determined by plotting inhibition percentages against sample concentrations and fitting the data to a nonlinear regression model using GraphPad Prism 9.0 software (GraphPad Software, San Diego, CA, USA).

### Antibacterial activity

The antibacterial activity of the synthesized compounds was evaluated following a procedure previously reported in our earlier studies, with minor modifications (Yıldırım et al. 2025b). Briefly, the compounds were diluted in Mueller–Hinton Broth (MHB) and transferred into 96-well microplates. Bacterial suspensions of carbapenem-resistant *A. baumannii* (BAA-1792), *E. coli* (BAA-2340), and *K. pneumoniae* (BAA-1705) were adjusted to a final inoculum density of 1.5 × 10⁶ CFU/mL and added to each well. After incubation under optimal growth conditions, minimum inhibitory concentrations (MICs) were determined using a resazurin-based colorimetric assay, where a color change from blue to pink indicated bacterial growth.

For minimum bactericidal concentration (MBC) determination, 10 µL aliquots were taken from wells showing no visible color change (i.e., no bacterial growth) and spread onto Mueller–Hinton agar plates. The plates were incubated for 24 h at 37 °C, and the lowest concentration of each compound that resulted in no visible bacterial colony formation was recorded as the MBC value. All experiments were performed in triplicate, and results were expressed as mean ± standard deviation (SD).

### Antibiofilm activity

To evaluate the antibiofilm effect of triphenylphosphonium-hydrazone derivatives (1a–1e), the bacterial suspensions (10^6^ CFU/mL) of the fresh cultures of carbapenem resistance *A. baumannii* and *E. coli* were added at 100 µL in 96 well plate, which containing Tryptic Soy Broth (TSB). The well plates were incubated at 37 °C for 24 h. Following incubation, the non-adherent bacteria of the wells was discarded and washed with phosphate-buffered saline (PBS). Two-fold serial dilutions of triphenylphosphonium-hydrazone derivatives (1a–1e), which prepared ranging from 1024 µg/mL to 16 µg/mL were added into the wells. The plates were incubated at 37 °C for 24 h. The assessment of antibiofilm activity by using crystal violet staining method was performed as it was previously described (Yıldırım et al. 2025c). The antibiofilm test was conducted triplicate.The dose-dependent antibiofilm percentages of triphenylphosphonium-hydrazone derivatives (1a–1e) was calculated with the following equation:

$$\left[\left.\left.\left(Control {OD}_{570} nm-Sample {OD}_{570} nm\right)/Control {OD}_{570} nm\right)\right)\right]\times 100$$

where *C* = absorbance of the control (biofilm, no treatment), and *S* = absorbance of the test (biofilm and treatment).

### Molecular docking

Molecular docking studies were conducted using Maestro 13.8 (Schrödinger Suite 2023–3) following our previously described protocol with minor adjustments (Yildirim et al. 2025). The three-dimensional structures of the target proteins were retrieved from the Protein Data Bank (PDB) and included: 4EY7 (acetylcholinesterase, *Torpedo californica*), 4JF4 (NDM-1 β-lactamase, *K. pneumoniae*), 2OV5 and 5UJ3 (KPC-2 β-lactamase, *K. pneumoniae*), 6MPQ (DNA gyrase subunit B, *Escherichia coli*), 6GIE (topoisomerase IV subunit B, *Acinetobacter baumannii*), 6OWS (penicillin-binding protein 1 A, *A. baumannii*), and 6PT1 (multidrug efflux pump AcrB, *E. coli*). The synthesized trisphosphonium hydrazone salts were drawn and energy-minimised using ChemDraw and LigPrep with the OPLS-4 force field. Docking simulations were performed using the Glide XP (extra-precision) algorithm, with binding affinities expressed in kcal/mol, where more negative values indicate stronger and more stable ligand–protein binding. The receptor grid was generated by selecting a representative atom within the active site of the co-crystallized ligand, after which a default grid box was automatically defined. For all target proteins, the grid dimensions were uniformly set to 20 × 20 × 20 Å to ensure consistent sampling of the binding site. Protein–ligand interactions such as hydrogen bonds, π–π stacking and hydrophobic contacts were analysed using Maestro’s Protein–Ligand Interaction Profiler and Discovery Studio Visualizer (Kumari and Kumar 2025b, 2025c).

### Statistical analysis

All experiments were performed in triplicate, and the results were expressed as mean ± standard deviation (SD). Statistical analyses were carried out using GraphPad Prism (version 9, GraphPad Software, San Diego, CA, USA). The differences among samples were evaluated by one-way analysis of variance (ANOVA) followed by Tukey’s post hoc test to determine significant differences between groups. Differences were considered statistically significant at *p* < 0.05. Distinct letters (a–e) in the tables indicate statistically different groups.

## Results and discussion

### Chemistry

Figures 1 and 2 depict the synthetic routes employed to obtain compound 1 and the tri-*p*-tolylphosphonium–hydrazone hybrid derivatives. Compound 1 was synthesized by refluxing tri-*p*-tolylphosphine with 4-(chloromethyl)benzaldehyde for 24 h. Subsequent reaction of compound 1 with the corresponding hydrazide derivatives in methanol under continuous stirring furnished the tri-*p*-tolylphosphonium–hydrazone hybrids. These transformations proceeded efficiently, affording seven novel compounds that were isolated by precipitation following the addition of diethyl ether. Structural elucidation of all products was carried out using comprehensive spectroscopic techniques, including ^1^H, ^13^C, and ^31^P NMR, along with HR-MS.

### Spectroscopic analysis of the compounds

In the ^1^H NMR spectra of the tri-*p*-toluylphosphonium-chalcone derivatives (1a–e), the methylene (-CH₂-) protons appeared as doublets at approximately 5.8 ppm, integrating for two protons. The methyl protons associated with the phosphine moiety were observed as singlets around 2.4 ppm, corresponding to a total integration of nine protons. The aromatic proton resonances were recorded within the range of 6.97–8.48 ppm for all derivatives. Additionally, the hydrazone NH proton exhibited a characteristic downfield signal at approximately 12 ppm, confirming the presence of the hydrazone functionality.

In the ^13^C NMR spectra of the tri-*p*-toluylphosphonium-chalcone hybrid salts, characteristic signals were observed at approximately 21 ppm, corresponding to the methyl carbons of the phosphine moiety. The methylene carbon (-CH₂-) resonances appeared around 55 ppm. The aromatic carbon signals were recorded within the range of 113.38–147.86 ppm for all compounds, consistent with the presence of multiple substituted aromatic rings. In addition, each derivative displayed a distinct carbonyl (C = O) resonance at approximately 160 ppm, confirming the chalcone framework within the molecular structure.

Furthermore, the mass spectrometric data obtained for all synthesized compounds were in good agreement with the expected molecular structures, thereby confirming their successful formation.

### AChE inhibition

The linear regression coefficients (R^2^ = 0.9732–0.9925) obtained for the AChE inhibition assays indicate a strong linear correlation between concentration and percentage inhibition for all tested samples. This confirms that the experimental data are reliable and that the inhibitory effect increases proportionally with concentration. The IC₅₀ values ranged between 4.832 and 7.515 µg/mL, suggesting that the tested samples exhibit moderate to high AChE inhibitory potential. Since lower IC₅₀ values correspond to stronger inhibition, the order of inhibitory potency among the samples can be expressed as follows: 1b (4.832 ± 0,001 µg/mL) > 1c (5.705 ± 0,002 µg/mL) >  1 d (6.204 ± 0,002 µg/mL) > 1a (6.729 ± 0,002 µg/mL) > 1e (7.515 ± 0,003 µg/mL) (Table 1).

**Table 1:** Linear regression equations, determination coefficients (R^2^), and IC₅₀ values for AChE inhibition of the samples 1a-1e

	equations	determination coefficients (R^2^)	IC_50_ (µg/mL)	IC_50_
1a	y = −5,9271x + 84,886	0,9732	6,729 ± 0,002^c,d^	11,8 ± 0,02^c,d^ µM
1b	y = −9,9628x + 115,86	0,9925	4,832 ± 0,001^a^	8,66 ± 0,01^a^ µM
1c	y = −8,6914x + 104,59	0,9864	5,705 ± 0,002^b^	9,19 ± 0,02^b^ µM
1d	y = −6,9486x + 90,61	0,9838	6,204 ± 0,002^c,d^	10,8 ± 0,02^c,d^ µM
1e	y = −5,7714x + 88,39	0,9903	7,515 ± 0,003^e^	13,9 ± 0,03^e^ µM

The strongest inhibition was observed for 1b, which likely exhibits a higher affinity toward the enzyme's active site compared to the others. Samples 1c also demonstrated considerable inhibitory activity, while 1a and 1e showed relatively weaker inhibition based on their higher IC₅₀ values. Overall, the consistently high R^2^ values (≥ 0.97) support the reliability of the experimental data and the suitability of the linear regression model. These findings indicate that 1b and 1c possess the most promising AChE inhibitory potential and may contain compounds with stronger interactions at the enzyme’s active site. The IC₅₀ values of the tested (1a–1e) revealed significant differences in their inhibitory activities. Among them, 1b exhibited the strongest inhibition with the lowest IC₅₀ value (8.66 ± 0.01 µM), which was statistically different from all other groups (*p* < 0.05). Sample 1c also showed a relatively strong activity (9.19 ± 0.02 µM), being slightly less potent than 1b but still significantly lower than the remaining samples. 1a and 1 d shared similar IC₅₀ values (11.8 ± 0.02 µM and 10.8 ± 0.02 µM, respectively) with no significant difference between them (*p* > 0.05). The weakest inhibitory effect was observed in 1e (13.9 ± 0.03 µM), which differed significantly from all other groups.

The determination coefficients (*R*^*2*^ = 0.973–0.993) indicated a strong linear correlation between concentration and inhibition percentage, confirming the reliability of the dose–response relationships. Overall, these findings suggest that the sample 1b possesses the most potent inhibitory capacity among the tested extracts, while 1e demonstrated the lowest activity.

**Fig. 3:**
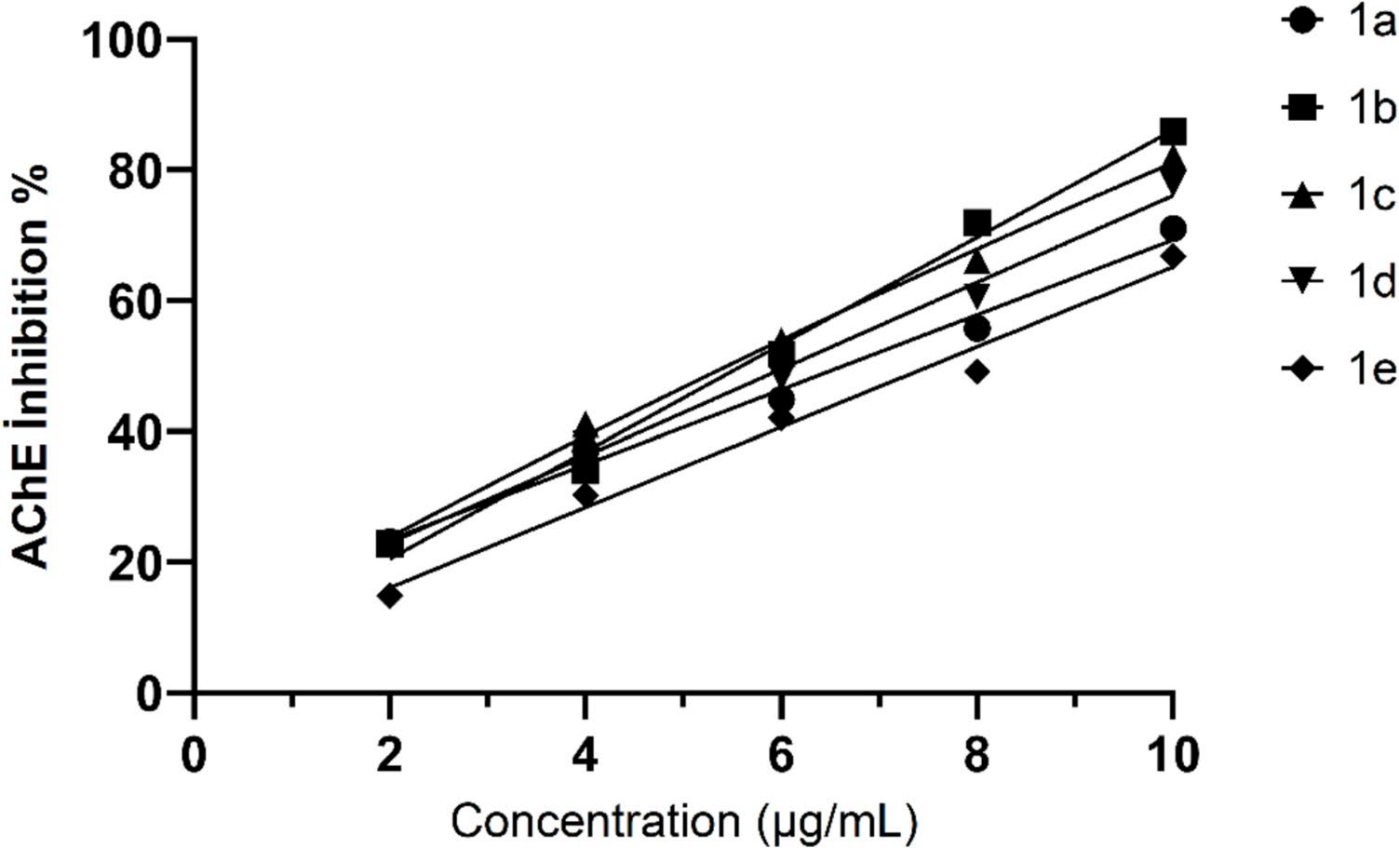
Dose-depented AChE inhibition of samples 1a-1e

All samples exhibited a clear dose-dependent increase in AChE inhibition with rising concentrations (2–10 µL). This indicates that enzyme inhibition was proportional to the applied sample volume. At the lowest concentration (2 µL), the highest inhibition was observed for samples 1c (23.09%) and 1b (22.83%), while sample 1e (14.89%) showed the weakest effect. At moderate concentrations (4–6 µL), samples 1c (41.27–53.63%) maintained strong inhibition levels, indicating a high affinity toward the AChE active site. At higher concentrations (8–10 µL), all samples exceeded 60% inhibition, with sample 1b (72–85.83%) and 1c (66.36–82.18%) showing the most potent effects. 1e consistently exhibited the lowest inhibition percentages (14.89–66.67%), suggesting a weaker interaction with the enzyme Fig. 3. Overall, samples 1b and 1c demonstrated the strongest inhibitory activity across all concentrations, confirming their potential as effective natural AChE inhibitors. Various hydrazone derivatives have been widely reported to exhibit potent AChE inhibitory activity, making them promising candidates for the development of anti-Alzheimer’s agents. Dincel et al. synthesized a series of hydrazide and hydrazone derivatives and demonstrated that these compounds possessed remarkable AChE inhibitory potency, with IC₅₀ values ranging from 4.91 to 36.47 nM (Dincel et al. 2024).

This strong inhibitory effect can be attributed to the structural characteristics of hydrazones, which typically contain both imine (–C = N–) and carbonyl functional groups capable of forming multiple interactions within the AChE active site (Yalazan et al. 2024; Kumar et al. 2024). Specifically, these groups can participate in hydrogen bonding, π–π stacking, and electrostatic interactions with key amino acid residues in the catalytic anionic site (CAS) and peripheral anionic site (PAS) of the enzyme. Such interactions hinder the hydrolysis of the neurotransmitter acetylcholine, leading to elevated acetylcholine levels in synaptic clefts (Wu et al. 2025; De Boer et al. 2021; Xu et al. 2017).

### Antibacterial activity

The MIC and MBC values of the synthesized compounds against carbapenem-resistant *A. baumannii***,** *E. coli*, and *K. pneumoniae* are presented in Table 2. The MIC values ranged from 32 to 256 µg/mL. Among the tested compounds, compound 1 d exhibited the most potent antibacterial activity, with MIC values of 32 µg/mL and 64 µg/mL against *A. baumannii* and *K. pneumoniae*, respectively. The remaining compounds displayed comparable inhibitory effects against *A. baumannii*, with MIC values of approximately 64 µg/mL. Overall, the synthesized compounds showed the lowest activity against *E. coli*. The enhanced antibacterial potency of compound 1 d may be attributed to the presence of the chlorine (Cl) substituent**,** which likely enhances its lipophilicity and facilitates stronger interactions with bacterial target proteins. Metelytsia et al. reported that alkyl triphenylphosphonium and alkyl tributylphosphonium bromides exhibited antimicrobial activity against multidrug-resistant *A. baumannii*, with MIC values ranging from 6.25 to 25.0 µM. In another study, a sterically hindered quaternary phosphonium salt was synthesized and evaluated against different isolates of *A. baumannii*, exhibiting MIC values of 4 and 8 µg/mL. In comparison, the compound showed an MIC value of 16 µg/mL against *K. pneumoniae* and 1–2 µg/mL against *E. coli* (Alfei et al. 2024*).* Hodyna et al. reported that 1,3-oxazol-4-yl phosphonium salt exhibited potent antibacterial activity against sensitive clinical isolates of *A.baumannii*, with MIC values ranging from ≤ 0.125 to 0.25 µg/mL (Hodyna et al. 2025).

**Table 2:** Antibacterial activity (MIC and MBC values, µg/mL) of triphenylphosphonium-hydrazone derivatives (1a–1e) against carbapenem-resistant bacterial strains compared with colistin

Bacterial strains	1a		1b		1c		1d		1e		Colistin	
	MIC	MBC	MIC	MBC	MIC	MBC	MIC	MBC	MIC	MBC	MIC	MBC
*A. baumannii*	64	128	128	256	64	128	32	64	64	128	2	4
*E. coli*	128	512	256	512	256	512	128	256	128	256	1	2
*K.pneumoniae*	256	512	256	512	128	256	64	256	256	512	0.5	1

### Antibiofilm activity

The antibiofilm activity of triphenylphosphonium-hydrazone derivatives (1a–1e) at concentration of 1024–16 µg/mL was assessed by using the crystal violet method towards *A. baumannii* and *E. coli*.

**Fig. 4:**
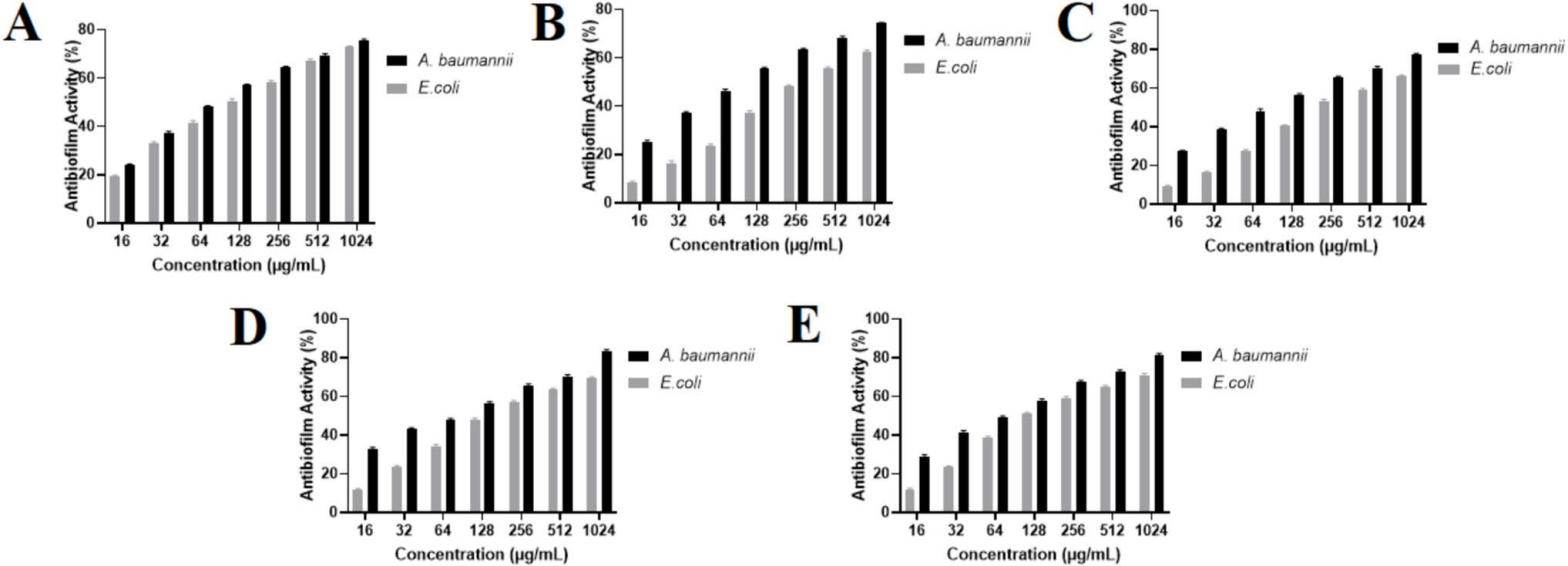
Antibiofilm activity of the synthesized compounds against *A. baumannii* and *E. coli*. (A–E) Dose-dependent inhibition of biofilm formation by compounds 1a (**A**)**,** 1b (**B**)**,** 1c (**C**)**,** 1 d (**D**) and 1e (**E**) at concentrations ranging from 16 to 1024 µg/mL. Black bars represent *A. baumannii* and grey bars represent *E. coli*. Data are presented as mean ± SD (n = 3)

Synthesized compounds exhibited the ability to inhibit microbial biofilms in a concentration-dependent manner, as shown in Fig. 4. At high (1024 µg/mL) concentration, for *A. baumannii*, triphenylphosphonium-hydrazone derivatives (1a–1e) disrupted substantial pre-formed biofilm by 75.6% ± 0.5, 74.4% ± 0.2, 77.1% ± 0.5, 83.4% ± 0.5, and 81.3% ± 0.8, in turn. On the other hand, the antibiofilm percentage of triphenylphosphonium-hydrazone derivatives (1a–1e) at low (16 µg/mL) concentration was detected as 24.1% ± 0.4, 25.2% ± 0.4, 27.2% ± 0.4, 32.4% ± 0.9, and 28.5% ± 1 µg/mL towards *A. baumannii*.

Against *E*. *coli*, triphenylphosphonium-hydrazone derivatives (1a–1e) demonstrated considerable biofilm eradication activity by 72.8% ± 0.5, 62.3% ± 0.6, 66.2% ± 0.4, 69.4% ± 0.4, and 71.1% ± 0.7 at 1024 µg/mL concentration, respectively. However, triphenylphosphonium-hydrazone derivatives (1a–1e) showed limited biofilm eradication rate of 19.3% ± 0.4, 8.2% ± 0.5, 9.4% ± 0.5, 11.5% ± 0.6, and 11.7% ± 0.6 against *E*. *coli* at 16 µg/mL concentration.

### Molecular docking

**Fig. 5:**
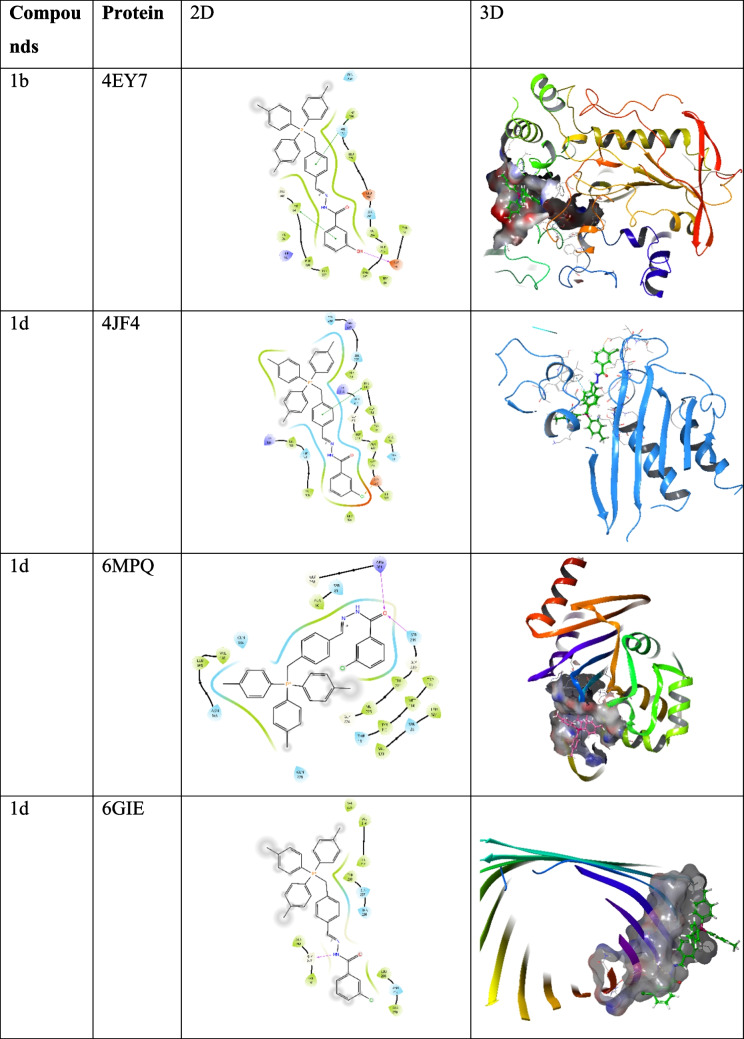

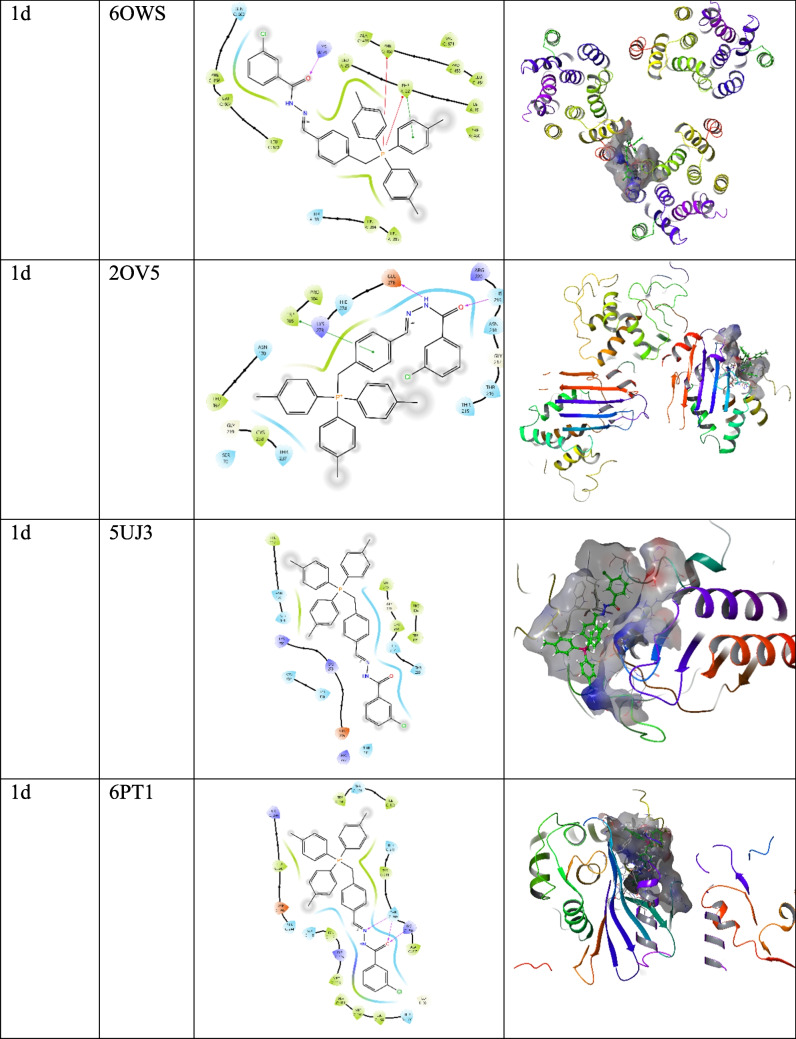
2D and 3D binding interactions of compounds in the active site of targeted proteins

To support the experimentally observed biological activities, molecular docking studies were performed. Among the synthesized compounds, compound 1b exhibited a strong binding affinity toward the AChE (PDB ID: 4EY7) enzyme, with a docking score of –8.096 kcal/mol**.** The aromatic rings of compound 2115 formed π–π stacking interactions with Trp286 and Tyr341, while the hydroxyl group established a hydrogen bond with Asp74**.** In antibacterial docking analyses, compound 1 d demonstrated the highest binding affinity, consistent with its strong experimental antibacterial activity. The docking scores of the tested compounds ranged from –4.091 to –6.662 kcal/mol, with the most stable interaction observed between 1 d and the penicillin-binding protein 1 A (PDB ID: 6OWS)**,** exhibiting a score of **–**6.662 kcal/mol (Table 3)**.** The carbonyl group of the compound formed a hydrogen bond with Lys29, while the aromatic moiety engaged in π–π stacking with Phe22 and cation–π interactions involving Phe22 and Phe458 through the phosphate group. These results suggest that the observed enzyme inhibitory and antibacterial effects are supported by strong and specific ligand–protein interactions at the molecular level (Fig. 5). Consistent with its superior MIC profile, compound 1 d showed the most favorable docking to PBP1A (6OWS; –6.662 kcal/mol), a key enzyme in peptidoglycan cross-linking, suggesting that disruption of cell-wall biosynthesis is a primary mechanism underlying its antibacterial activity. A secondary but notable affinity toward the AcrB efflux pump (6PT1; –6.231 kcal/mol) may further enhance intracellular exposure by attenuating active drug efflux. Together, these interactions provide a mechanistic rationale for the lowest MICs observed for 1 d against *A. baumannii* and *K. pneumoniae*. While docking scores alone cannot prove causality, the agreement between binding predictions and phenotypic readouts strengthens the structure–activity relationship and motivates targeted validation (e.g., PBP1A transpeptidase assays, efflux-deficient strains, and uptake/accumulation studies).

**Table 3:** Molecular docking scores and Glide emodel values of triphenylphosphonium-hydrazone derivatives

Compounds	Protein	Docking score	Glide emodel
1b	4EY7	−8.096	−86.922
1d	4JF4	−4.091	−55.250
1d	6MPQ	−4.273	−50.266
1d	6GIE	−4.395	−41.422
1d	6OWS	−6.662	−59.328
1d	2OV5	−4.116	−49.070
1d	5UJ3	−4.966	−58.183
1d	6PT1	−6.231	−69.220

### Structure–Activity Relationship (SAR) of potent compounds

The biological activity of the synthesized compounds arises from the combined effects of the triphenylphosphonium (TPP⁺) cation, hydrazone linker, and aromatic substituents, which collectively regulate lipophilicity, reactivity, and target binding. The TPP⁺ moiety facilitates efficient membrane permeation and preferential mitochondrial accumulation due to the negative mitochondrial membrane potential, thereby enhancing mitochondria-targeted anticancer and antimicrobial effects. Increased phenyl ring lipophilicity improves cellular uptake; however, excessive lipophilicity may induce nonspecific toxicity, indicating the need for an optimal balance. The hydrazone linkage (–C = N–NH–) acts as a key pharmacophore capable of hydrogen bonding and dipole interactions with biological targets. Its chemical reactivity, including susceptibility to hydrolytic or redox processes, contributes to biological potency but may compromise stability at higher reactivity levels. Substituent effects on the aromatic ring strongly influence activity. Electron-withdrawing groups increase the electrophilicity of the hydrazone carbon, enhancing interactions with nucleophilic biological targets. Accordingly, chloro-substituted derivatives exhibited superior antibacterial activity compared to other analogues (Xie et al. 2019; Yan et al. 2024). In contrast, AChE inhibition was enhanced by hydroxyl (–OH) substituents, likely due to favorable hydrogen-bonding interactions within the enzyme active or peripheral site. Overall, these findings demonstrate that antibacterial and AChE inhibitory activities follow distinct SAR trends, emphasizing the importance of rational substituent selection for target-specific optimization.

## Conclusion

Five triphenylphosphonium-hydrazone derivatives (1a–1e) were successfully synthesized and structurally characterized using FT-IR, NMR, and HR-MS/MS analyses. All tested samples exhibited notable AChE inhibitory activity, with their IC₅₀ and R^2^ values confirming the reproducibility and reliability of the bioassay results. Among them, compound 1b demonstrated the most potent AChE inhibition, suggesting its strong potential as a promising natural inhibitor for managing neurodegenerative diseases such as Alzheimer’s disease.

Furthermore, compound 1 d exhibited the highest antibacterial activity, with MIC of 32 µg/mL against *A. baumannii* and 64 µg/mL against *K. pneumoniae*. Molecular docking analyses supported these findings, revealing the strongest binding affinity of 1 d to PBP1A (PDB: 6OWS) and a secondary interaction with AcrB (PDB: 6PT1), indicating a possible dual mechanism involving both cell-wall disruption and efflux pump inhibition.

Importantly, all derivatives (1a–1e) also demonstrated dose-dependent antibiofilm activity toward *A. baumannii* and *E. coli*, achieving up to 83.4% and 72.8% biofilm disruption at 1024 µg/mL, respectively, further supporting their multifunctional antimicrobial potential.

From a future perspective, the results of this study provide a solid foundation for the rational optimization of triphenylphosphonium-hydrazone scaffolds to enhance their potency, selectivity, and pharmacokinetic properties. Future work will focus on detailed cytocompatibility and toxicity evaluations in mammalian cell models, and in vivo validation of the most promising candidates. In addition, the multifunctional nature of these compounds opens new opportunities for the development of dual-action therapeutics targeting both neurodegenerative disorders and multidrug-resistant bacterial infections, particularly in clinical scenarios where infection-related neuroinflammation may play a contributory role.

## Supplementary Information

Below is the link to the electronic supplementary material.

Supplementary file1 (DOCX 919 KB)

## Data Availability

All source data for this work (or generated in this study) are available upon reasonable request.
