# Supplementary material for: Novel triphenylphosphonium-hydrazone salts: integrated experimental and computational insights into AChE inhibition and resistance-overcoming antimicrobial and antibiofilm potential
Source: Naunyn Schmiedebergs Arch Pharmacol. 2026 Jan 20;399(6):9189–201. doi: 10.1007/s00210-026-05002-8 (PMC13086752; doi:10.1007/s00210-026-05002-8)
Supplement: Supplementary file 1 — Supplementary file1 (DOCX 919 KB) [file 210_2026_5002_MOESM1_ESM.docx]

**Novel Triphenylphosphonium-Hydrazone Salts: Integrated Experimental and Computational Insights into AChE Inhibition and Resistance-Overcoming Antimicrobial and Antibiofilm Potential**

Metin Yıldırım^1,*^, Hakan Ünver^2^, Adem Necip^3^, Büsra Hord^4^, Mehmet Ersatir^5^

^1^Department of Biochemistry, Faculty of Pharmacy, Harran University, Sanliurfa, Türkiye

^2^Department of Chemistry, Faculty of Science, Eskisehir Technical University, Eskisehir, Türkiye

^3^Department of Pharmacy Services, Vocational School of Health Services, Harran University, Sanliurfa, Türkiye

^4^Faculty of Pharmacy, Harran University, Sanliurfa, Türkiye

^5^Department of Chemistry, Faculty of Art and Science, Cukurova University, Adana 01330, Türkiye

*Corresponding author: [metinyildirim4@gmail.com](mailto:metinyildirim4@gmail.com)


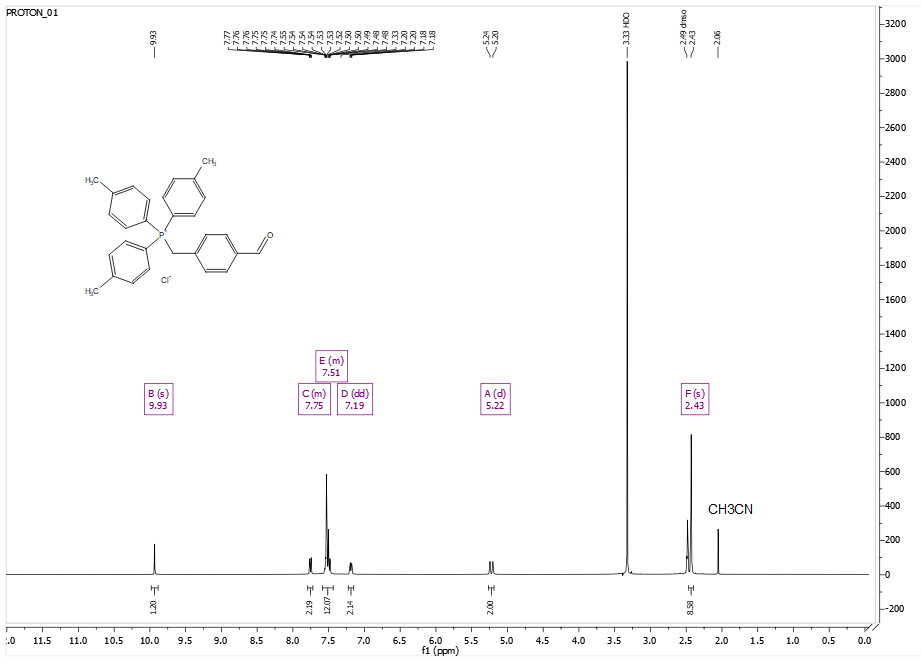


**Figure S1.** ^1^H-NMR spectra of compound **1**


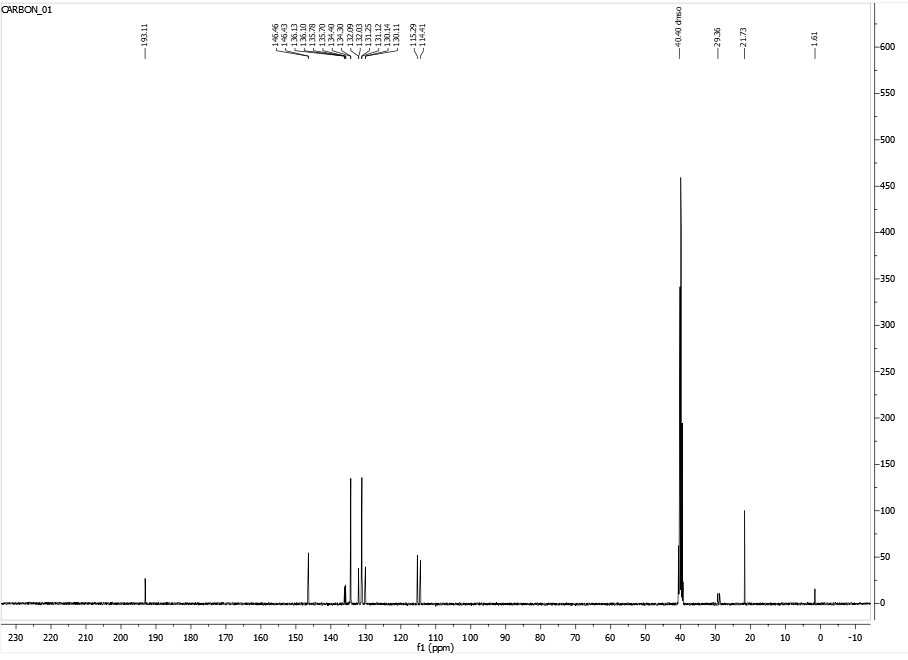


**Figure S2.**^13^C-NMR spectra of compound 1


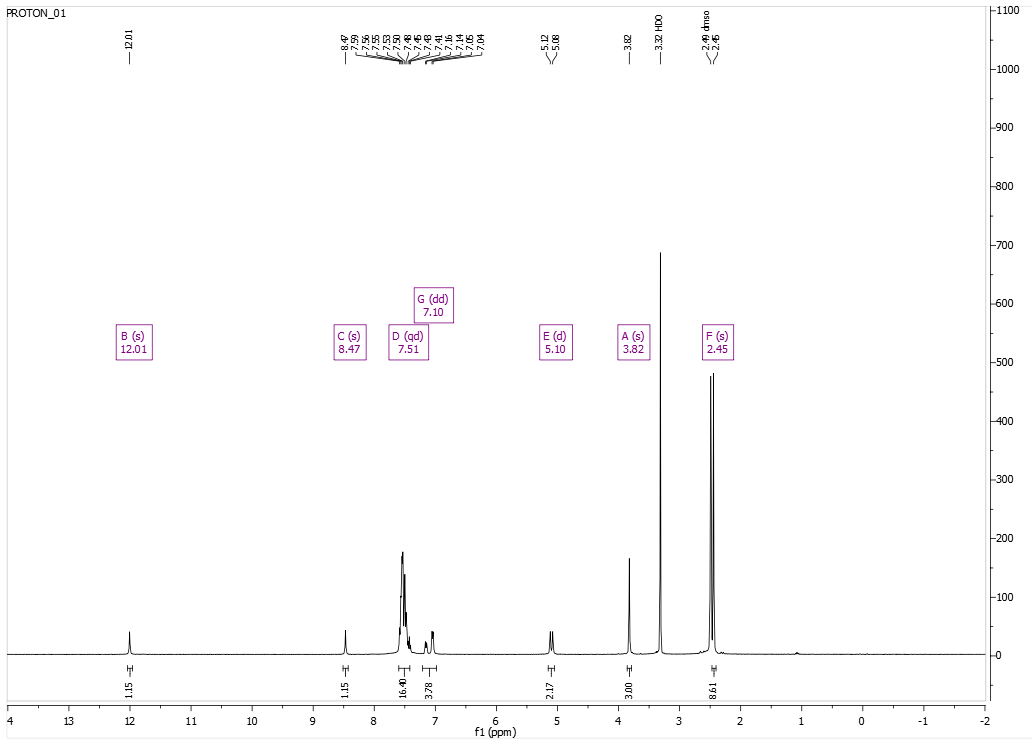


**Figure S3.** ^1^H-NMR spectra of compound **1a**


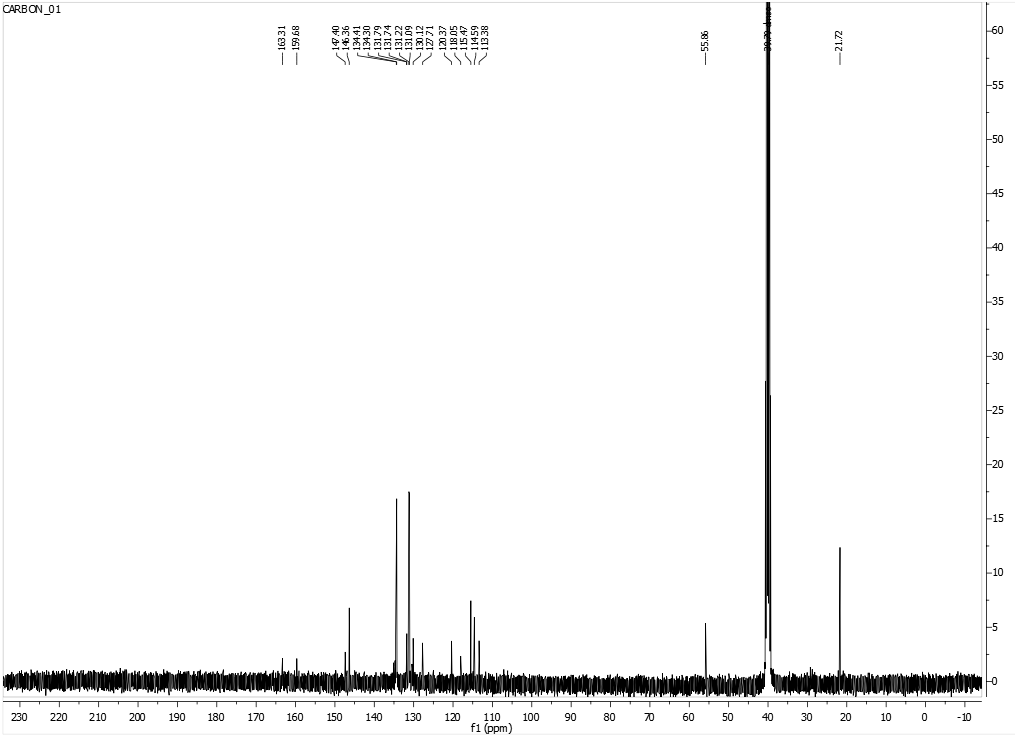


**Figure S4.**^13^C-NMR spectra of compound **1a**


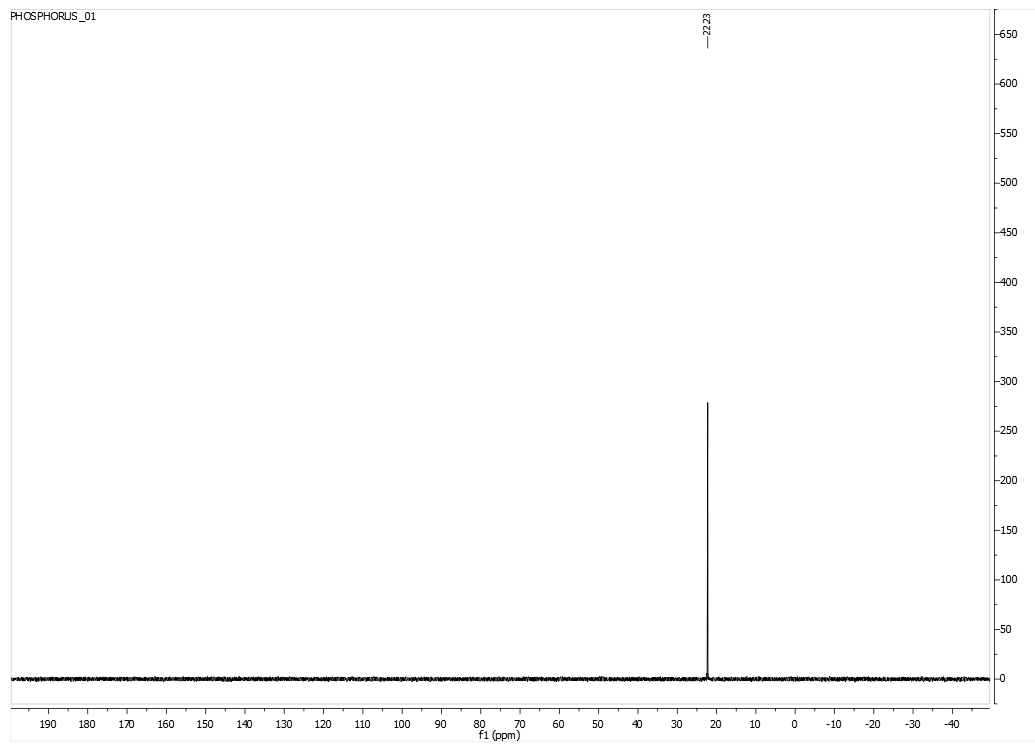


**Figure S5.**^31^P NMR spectra of compound **1a**


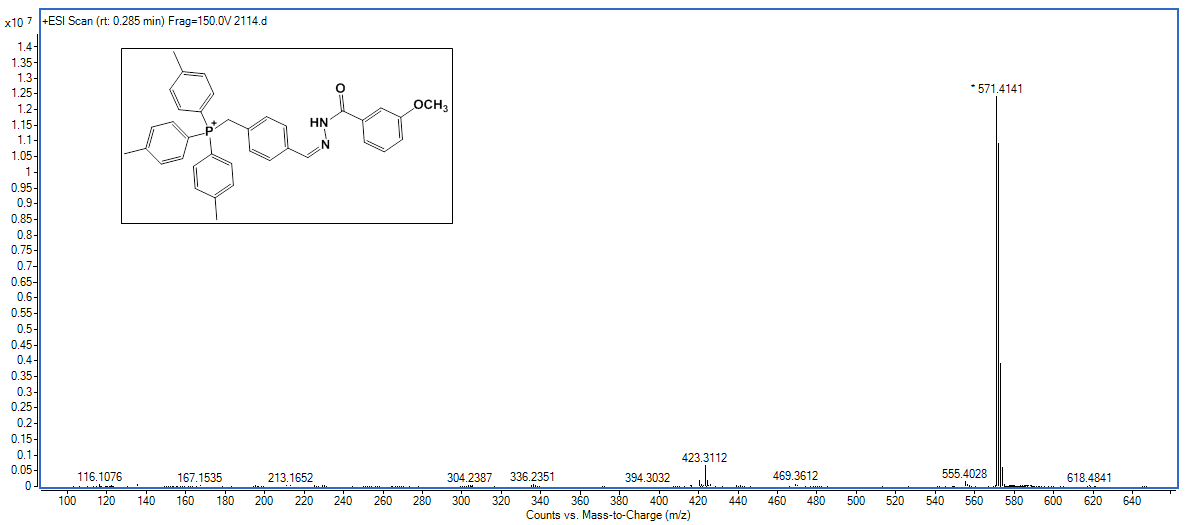


**Figure S6.** HR-ESIMS spectrum of compound **1a**


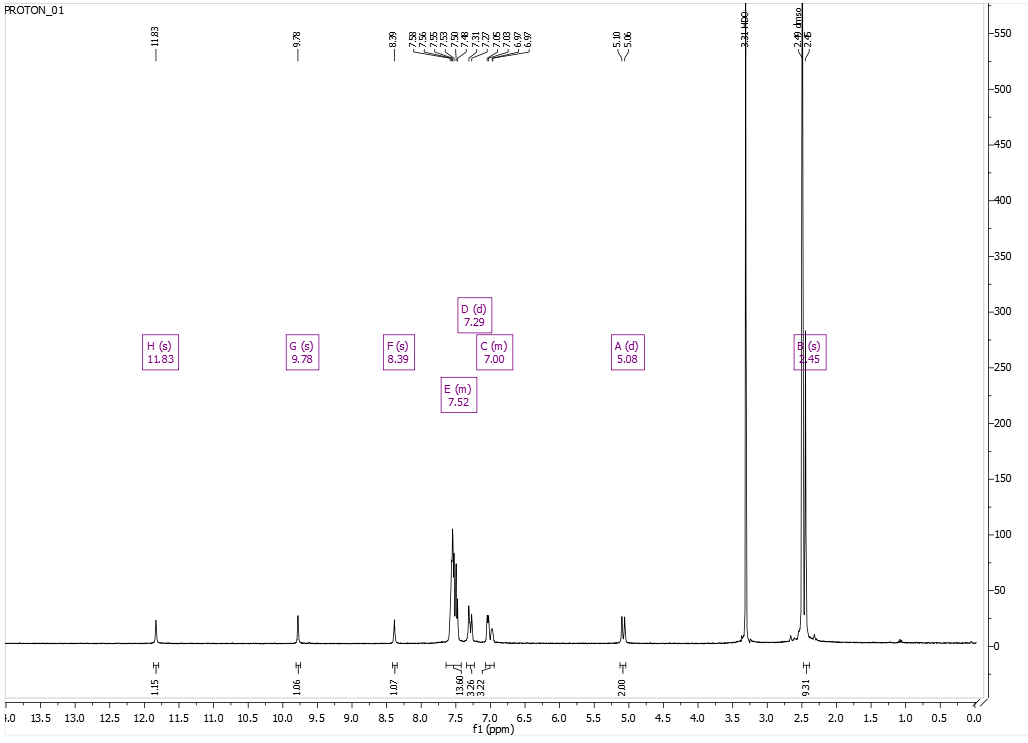


**Figure S7.** ^1^H-NMR spectra of compound **1b**


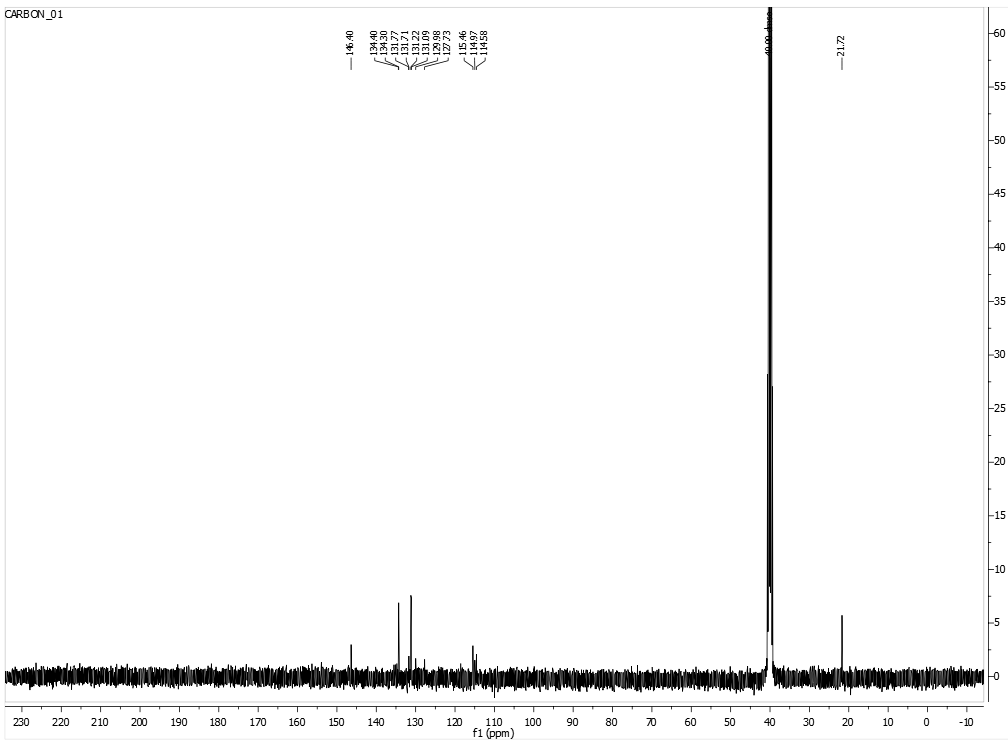


**Figure S8.**^13^C-NMR spectra of compound **1b**


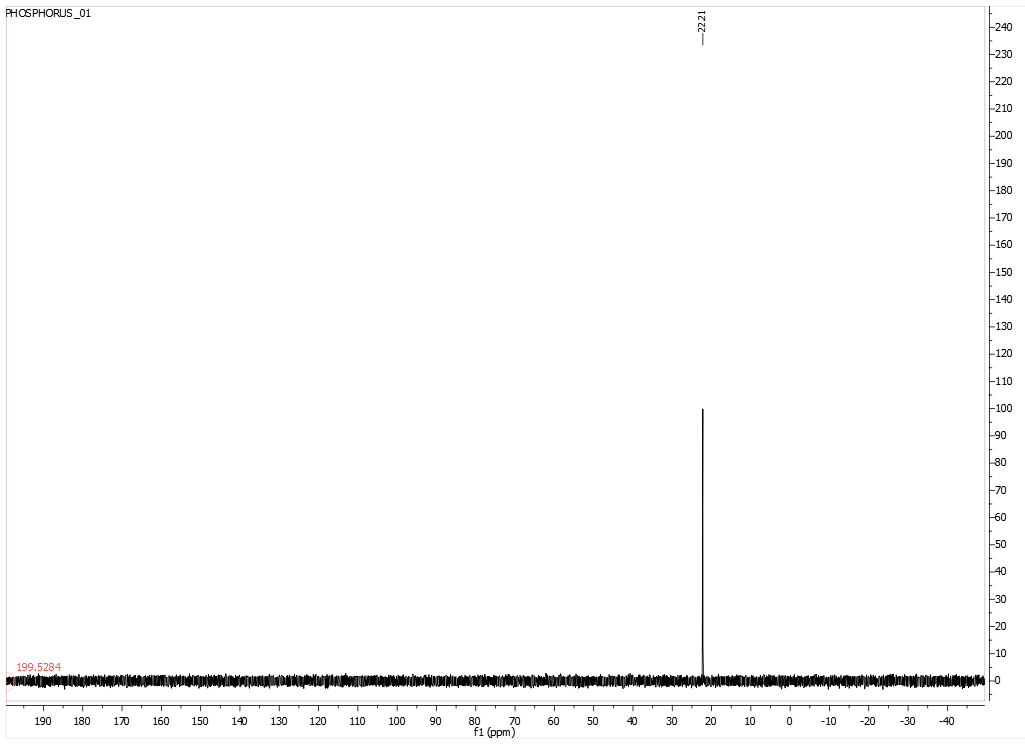


**Figure S9.**^31^P-NMR spectra of compound **1b**


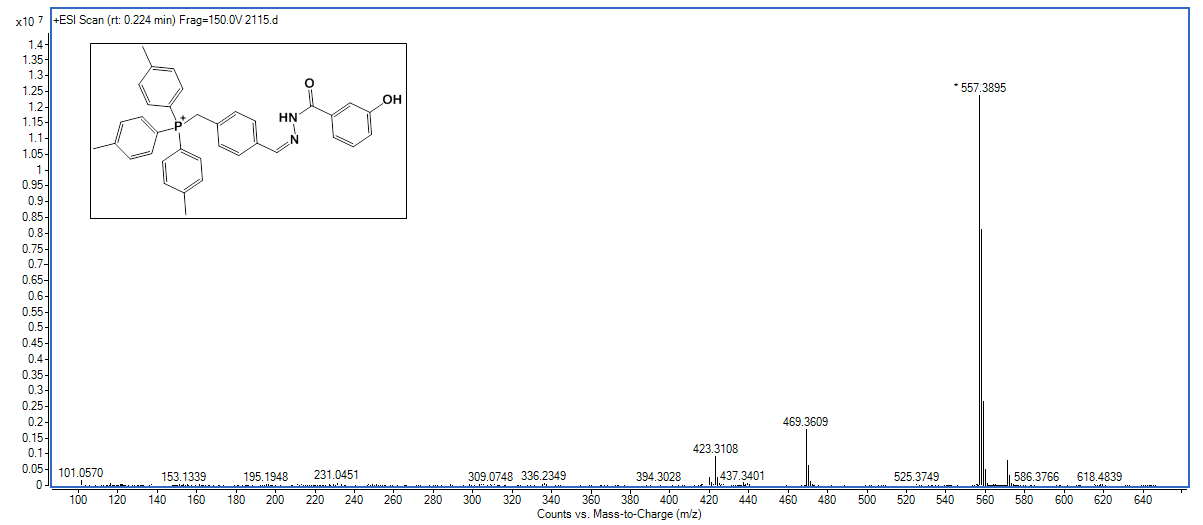


**Figure S10.** HR-ESIMS spectrum of compound **1b**


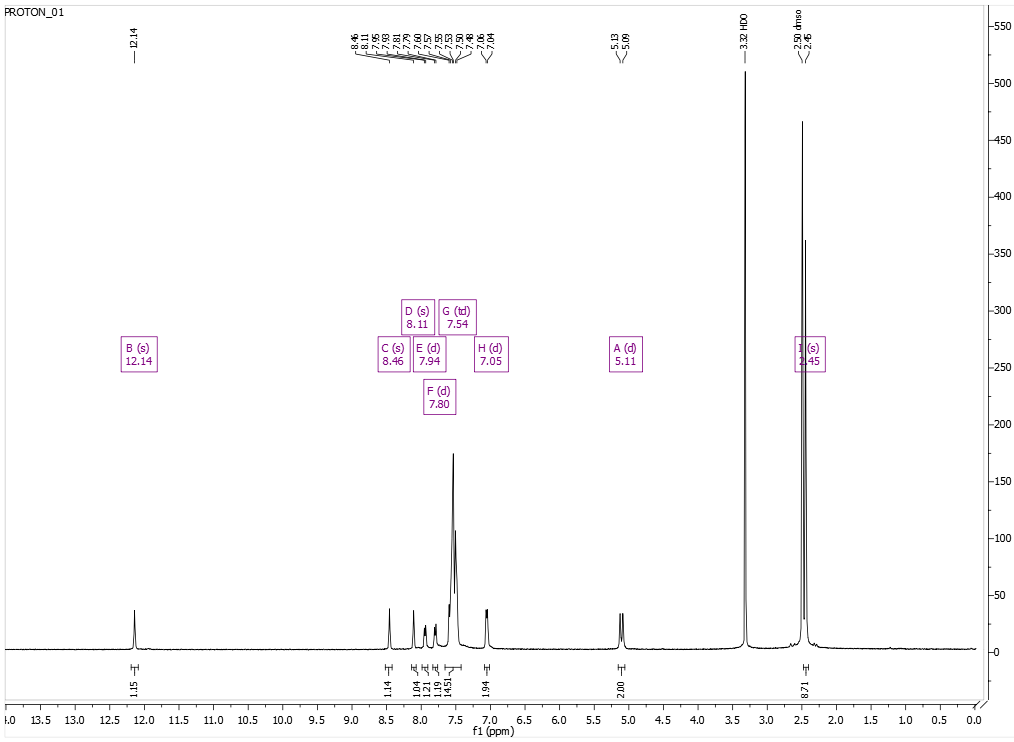


**Figure S11.** ^1^H-NMR spectra of compound **1c**


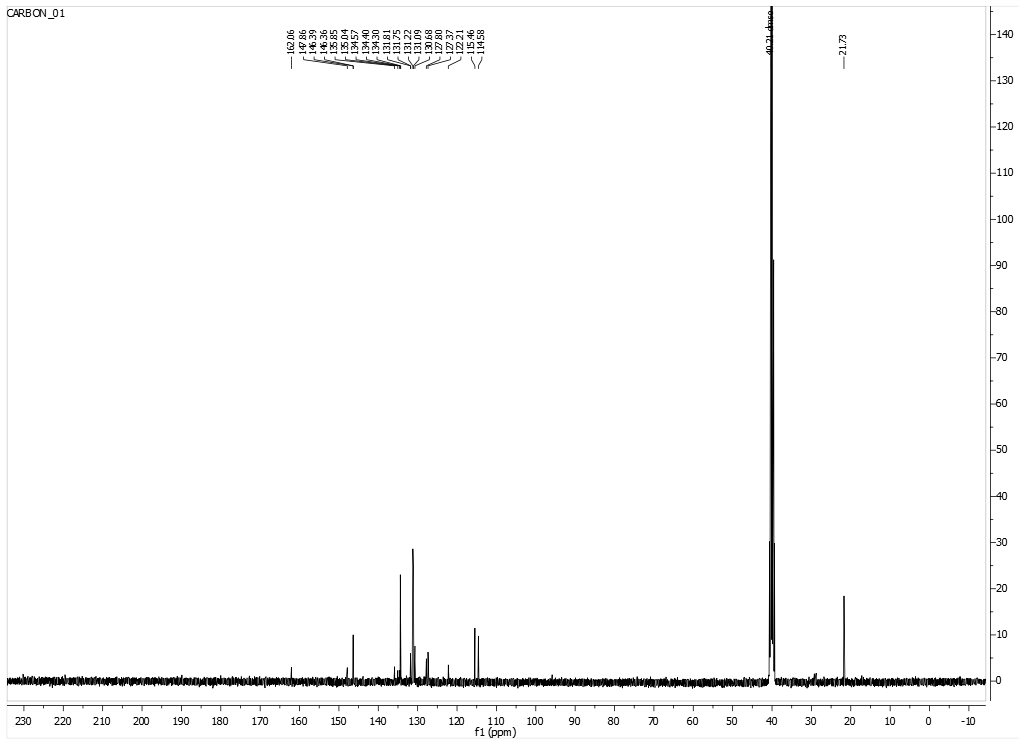


**Figure S12.**^13^C-NMR spectra of compound **1c**


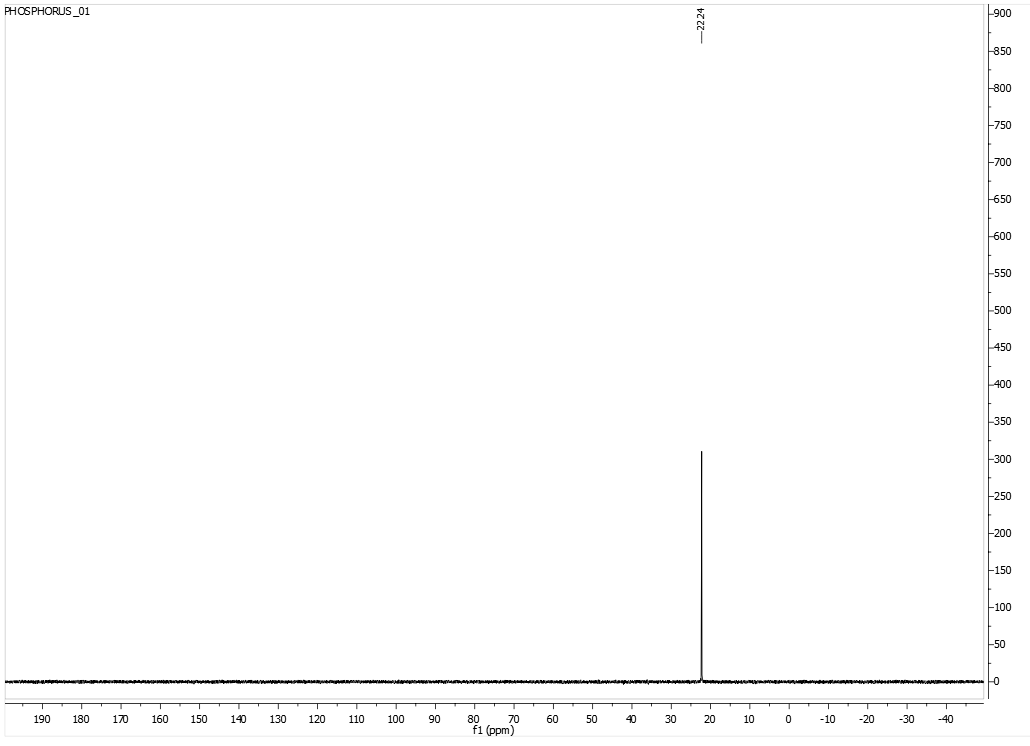


**Figure S13.**^31^P-NMR spectra of compound **1c**


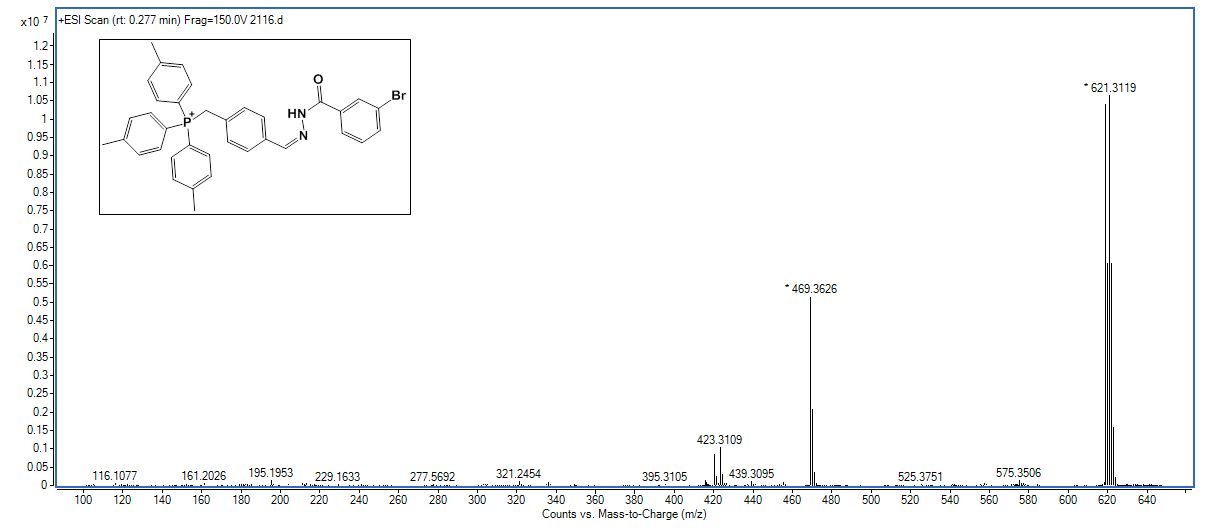


**Figure S14.** HR-ESIMS spectrum of compound **1c**


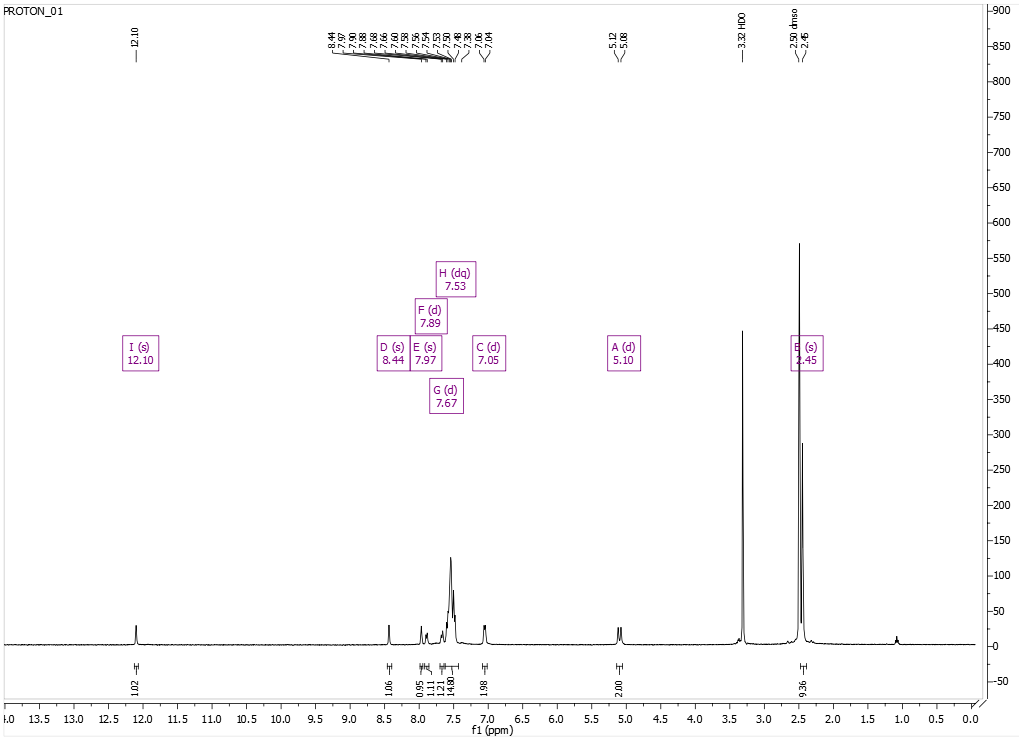


**Figure S15.** ^1^H-NMR spectra of compound **1d**


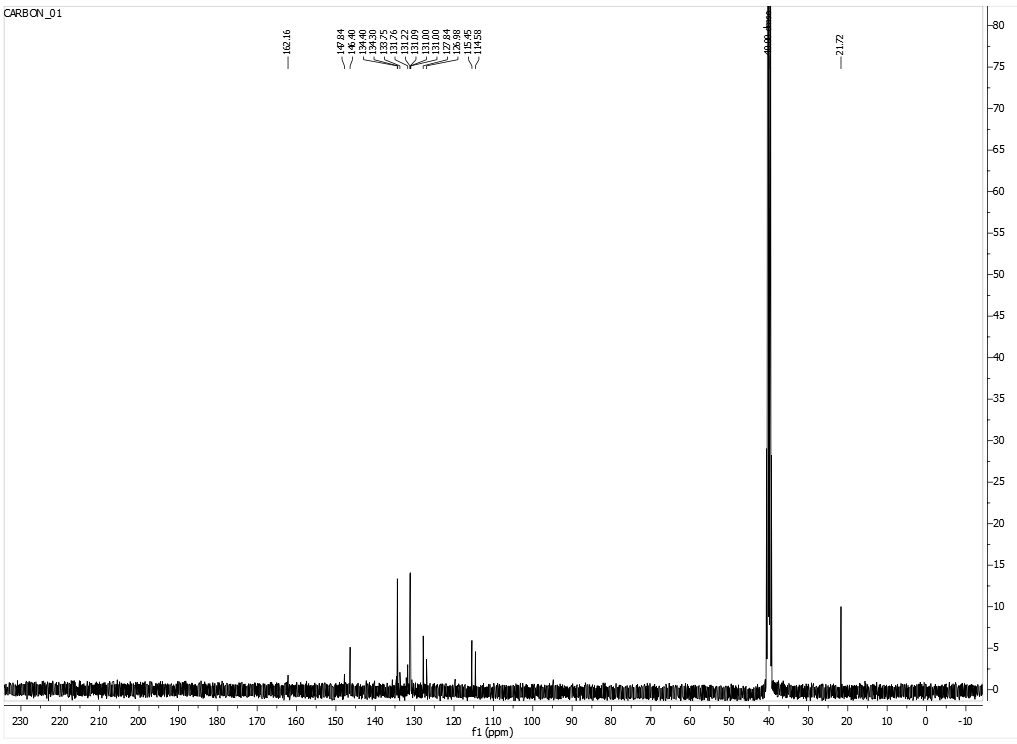


**Figure S16.**^13^C-NMR spectra of compound **1d**


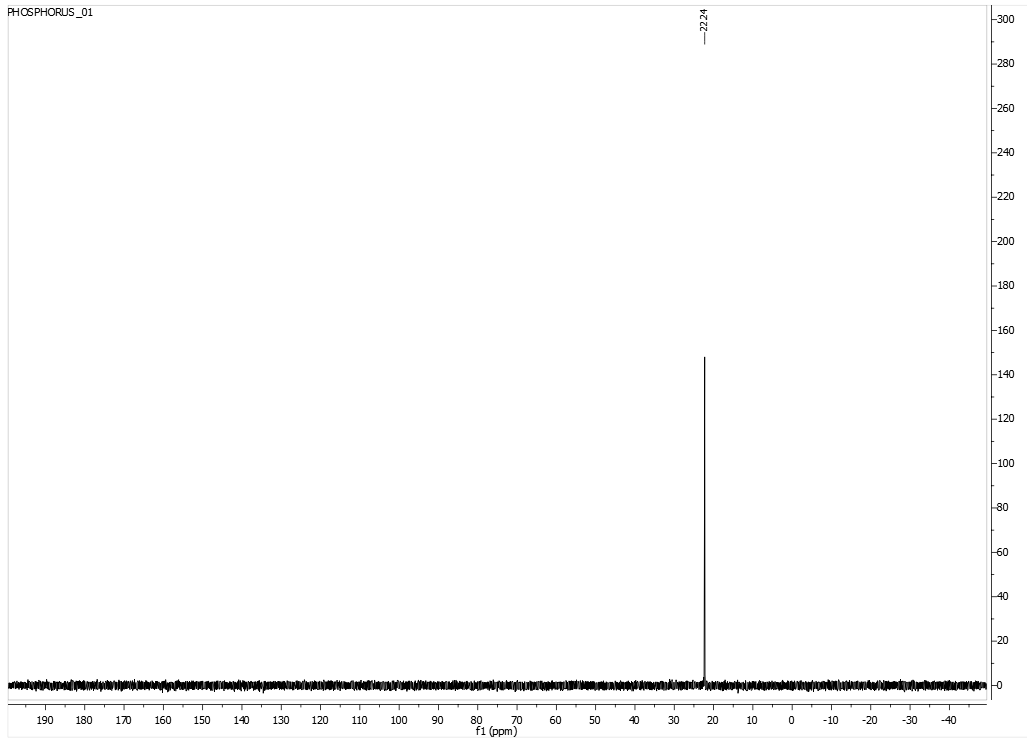


**Figure S17.**^31^P-NMR spectra of compound **1d**


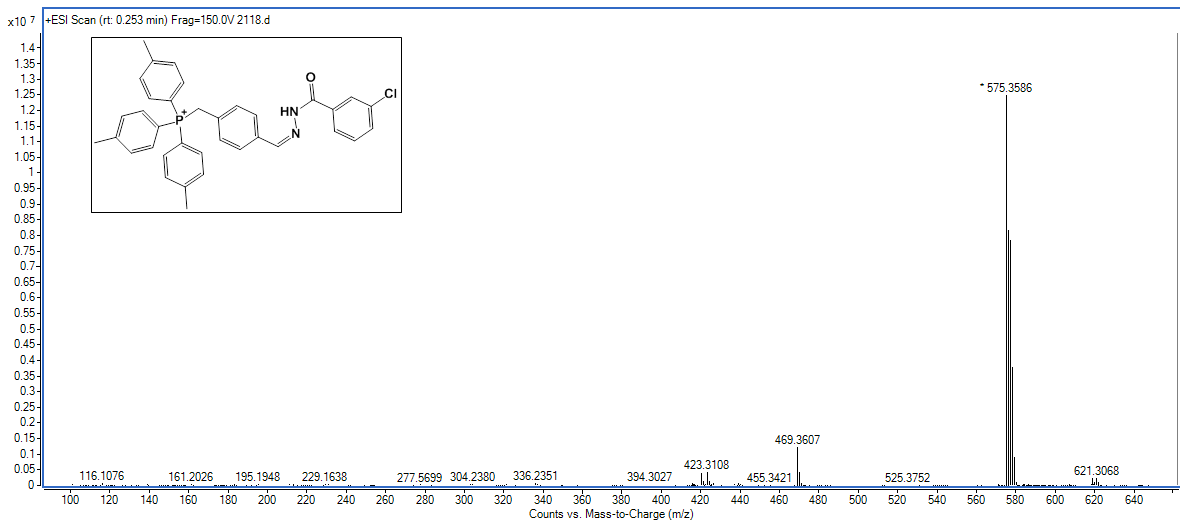


**Figure S18.** HR-ESIMS spectrum of compound **1d**


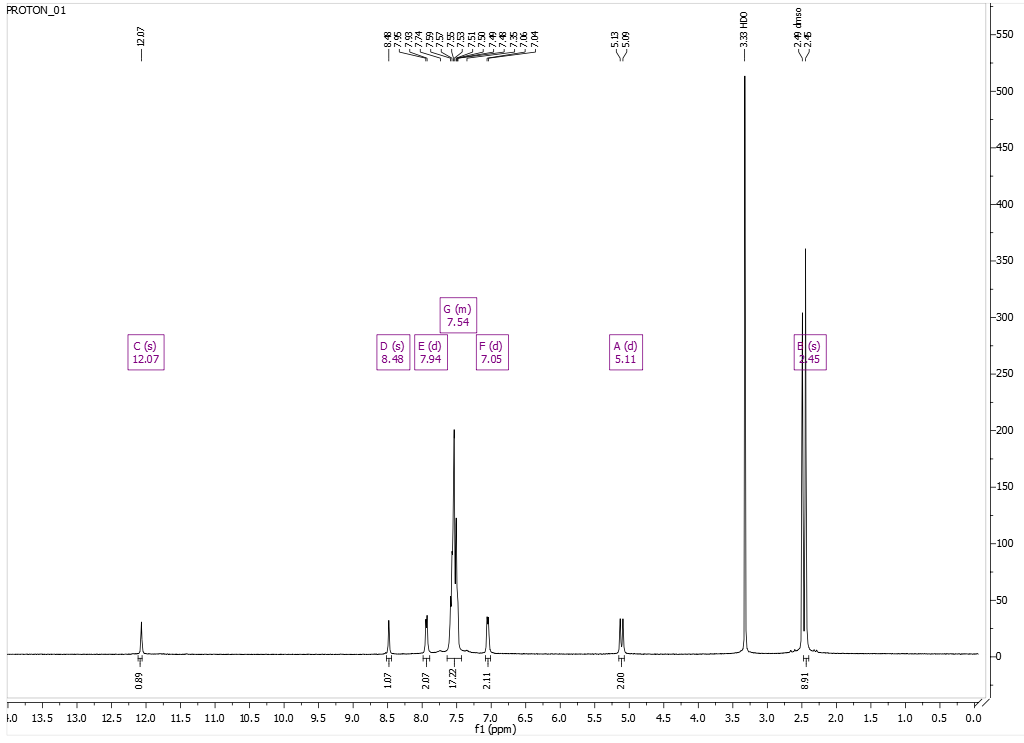


**Figure S19.** ^1^H-NMR spectra of compound **1e**


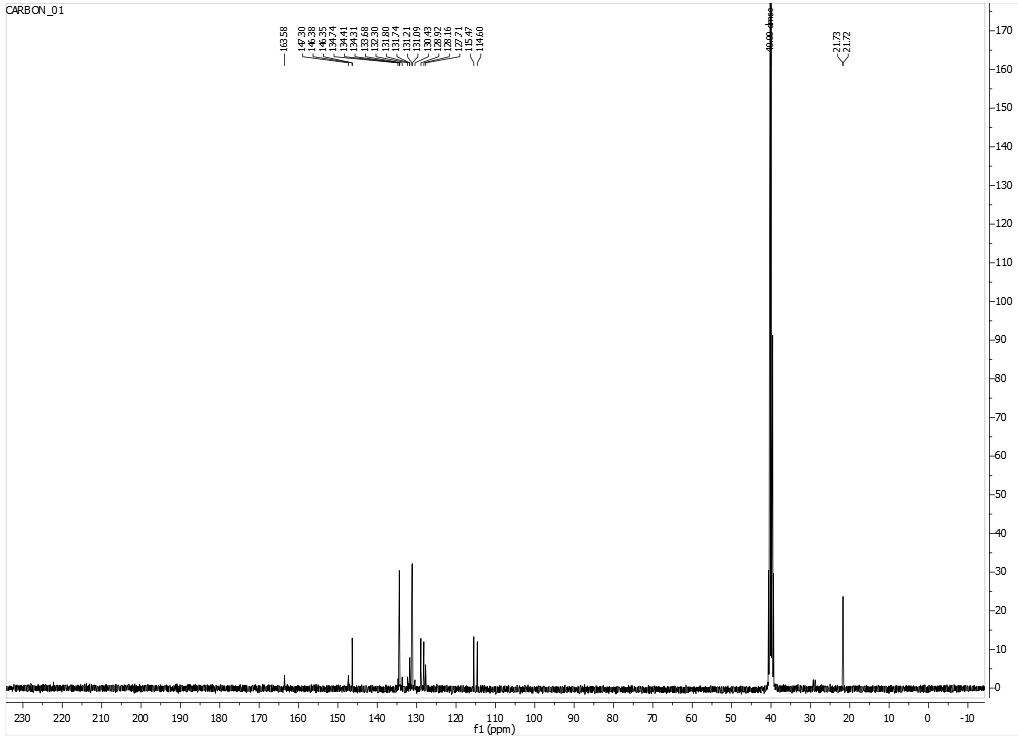


**Figure S20.**^13^C-NMR spectra of compound **1e**


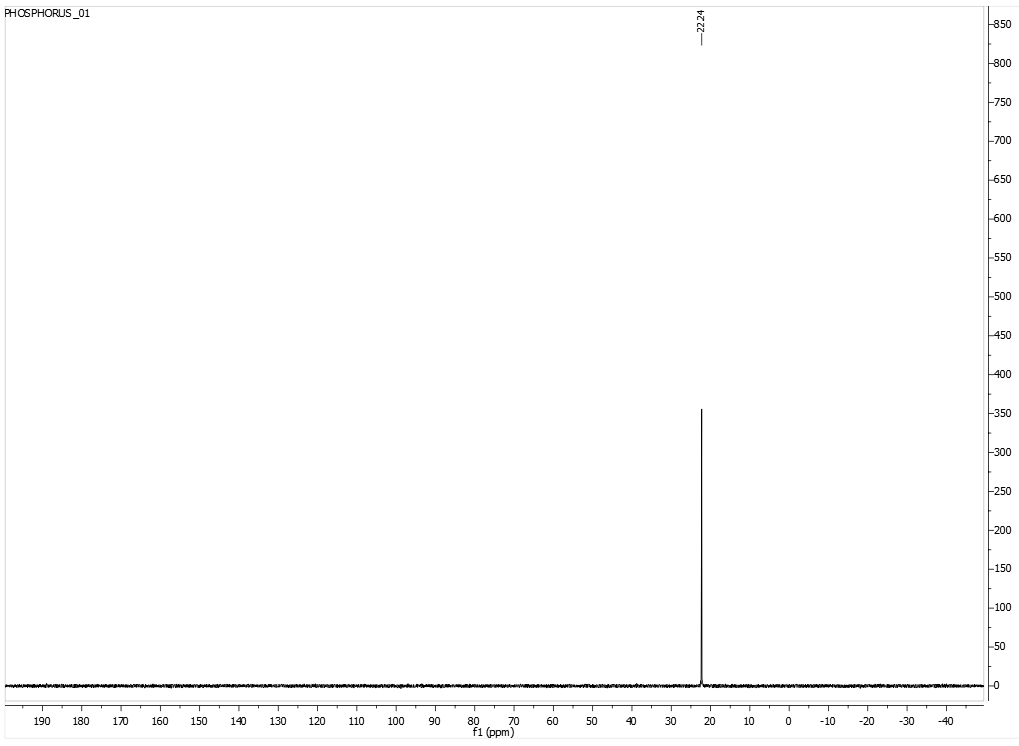


**Figure S21.**^31^P-NMR spectra of compound **1e**


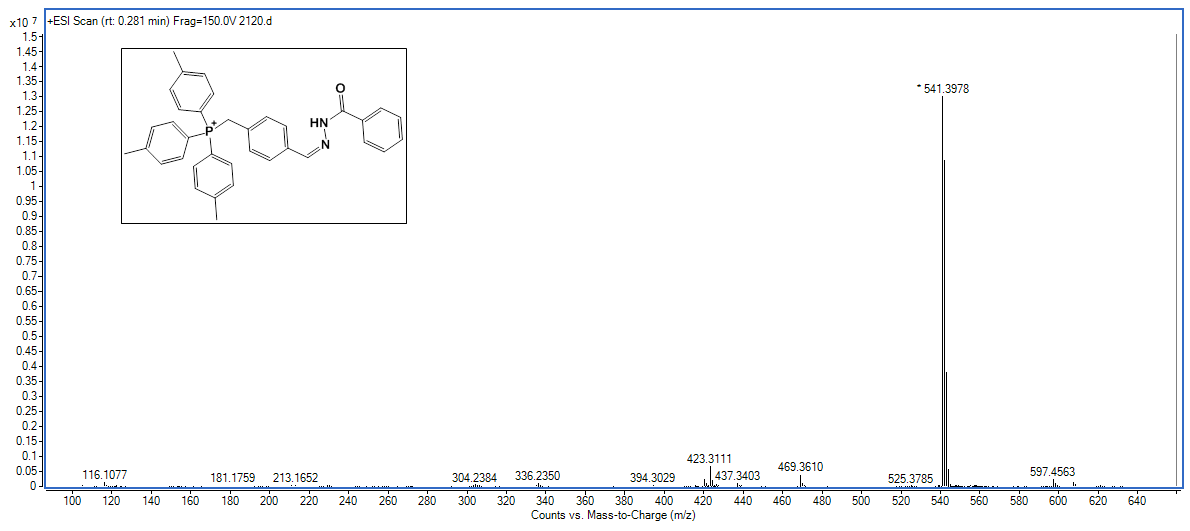


**Figure S22.** HR-ESIMS spectrum of compound **1e**
